# Supplementary material for: Environmental variation and the evolution of large brains in birds
Source: Nat Commun. 2016 Dec 22;7:13971. doi: 10.1038/ncomms13971 (PMC5192215; doi:10.1038/ncomms13971)
Supplement: Supplementary Information — Supplementary Figures, Supplementary Tables, Supplementary Methods and Supplementary References [file ncomms13971-s1.pdf]

Supplementary Figures

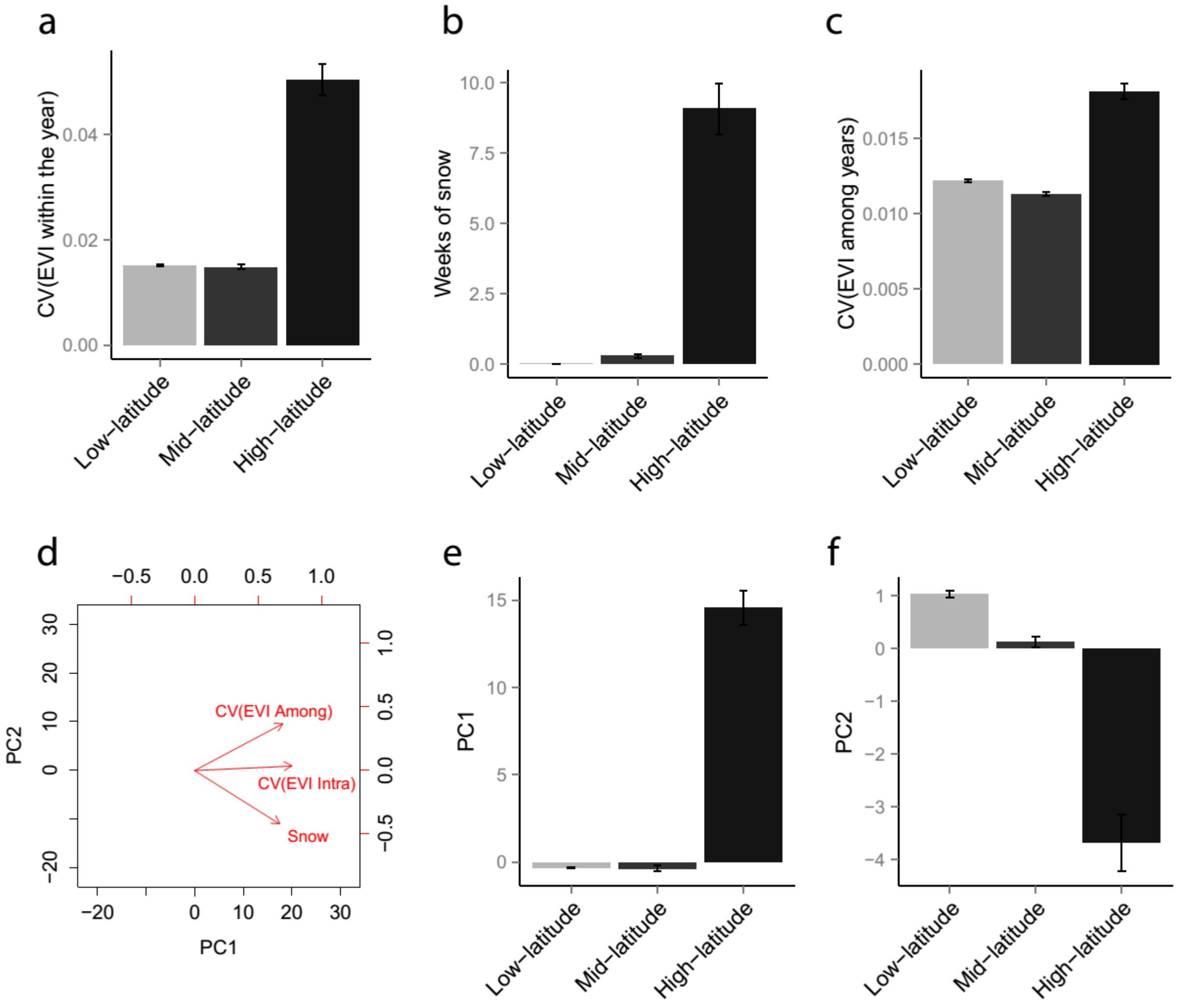

Supplementary Figure 1. Differences in enhanced vegetation index (EVI) variation within and among years and weeks of snow in the breeding site of resident birds at high (N=50), mid (N=326) and low (N=459) latitudes (**a-c**). Phylogenetic PCA (PPCA) with the three variables (**d**) and their co-variation with latitude (**e-f**). In all the bar plots the mean±SEM of the corresponding environmental factor is shown. Residents from higher, mid and low latitudes are represented by black, dark-grey and light-grey bars, respectively.

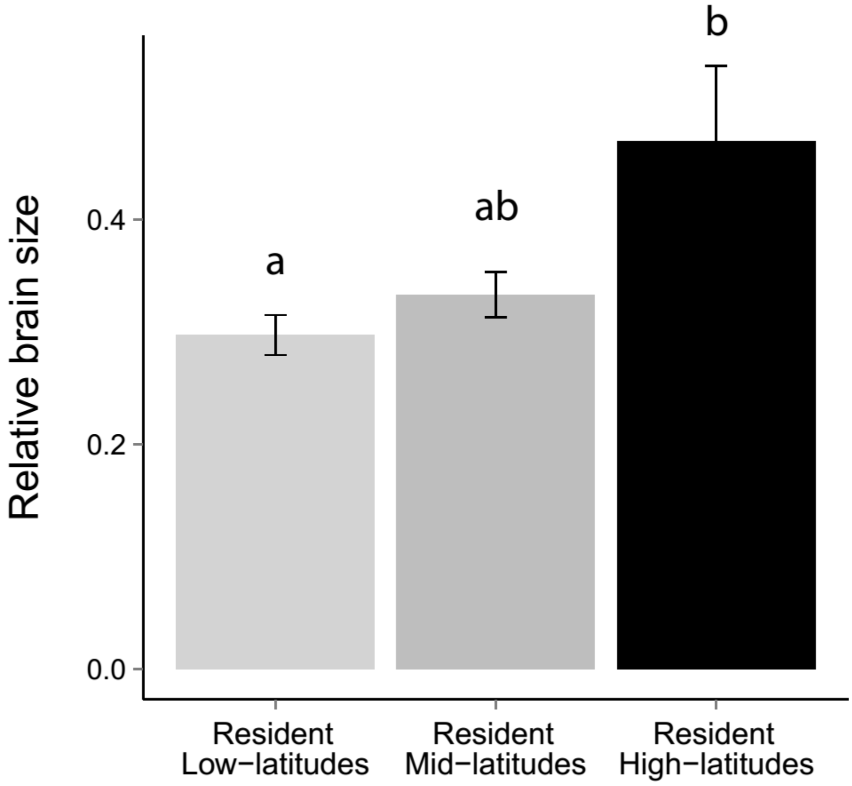

Supplementary Figure 2. Relative brain size (Mean ± SEM) in resident species as a function of latitude of the region where they occur. Residents from Higher latitude have larger brains than residents from lower latitudes (PGLS: $p = 0.015$, N=855, See Supplementary Table 1). Residents from higher (N=53), mid (N=335) and low (N=467) latitudes are represented by black, dark-grey and light-grey bars, respectively.

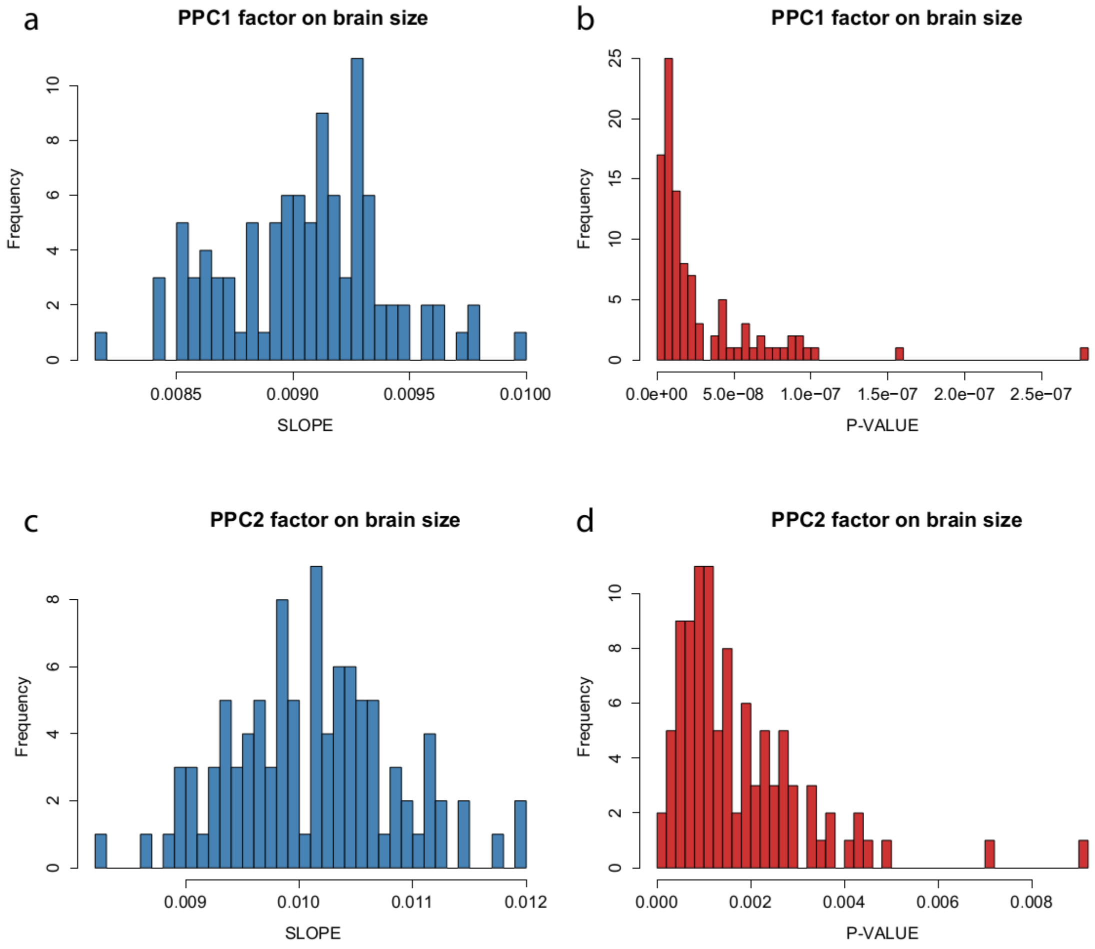

Supplementary Figure 3. Distribution of slopes (blue histograms) and p-values (red histograms) of PGLS models using 100 different phylogenetic trees linking brain size with PPC1 (**a-b**) and PPC2 (**c-d**), while controlling for log(body size).

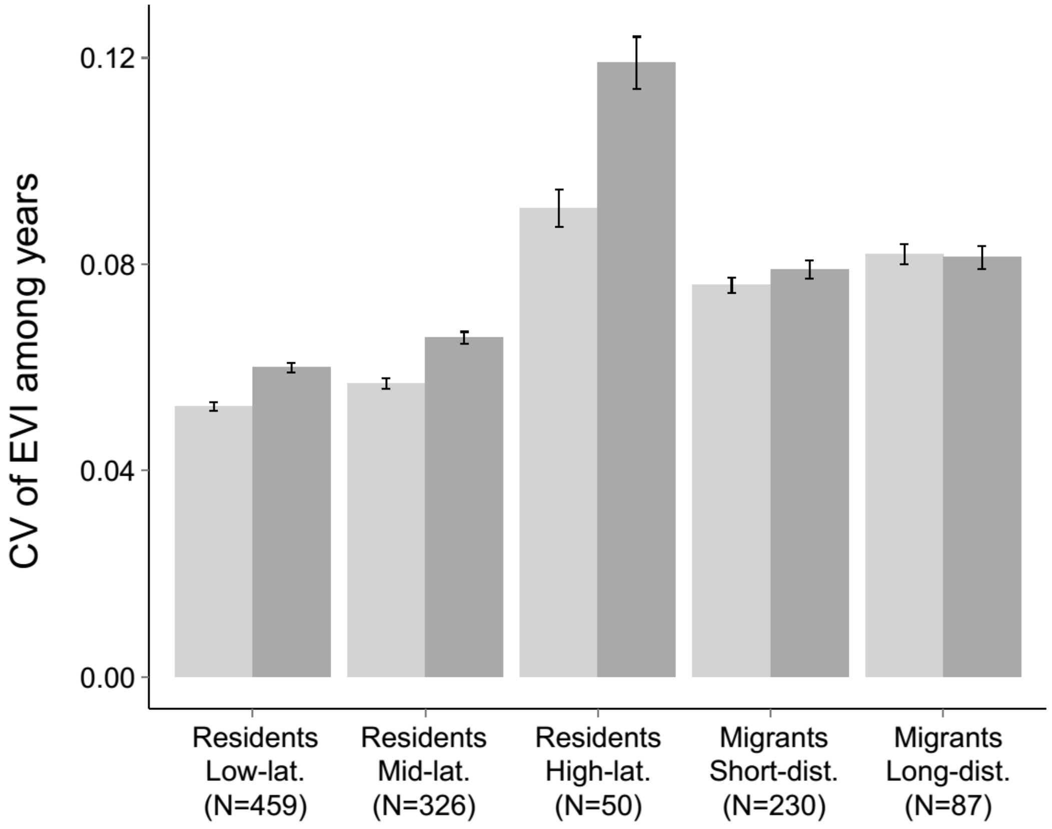

Supplementary Figure 4. Variation in productivity (Mean ± SEM), measured by the coefficient of variation of enhanced vegetation index (EVI) across 15 years during breeding season in the breeding distribution range (light grey) and during non-breeding season in the non-breeding distribution range (dark grey) of bird species exposed to different degree of environmental variation.

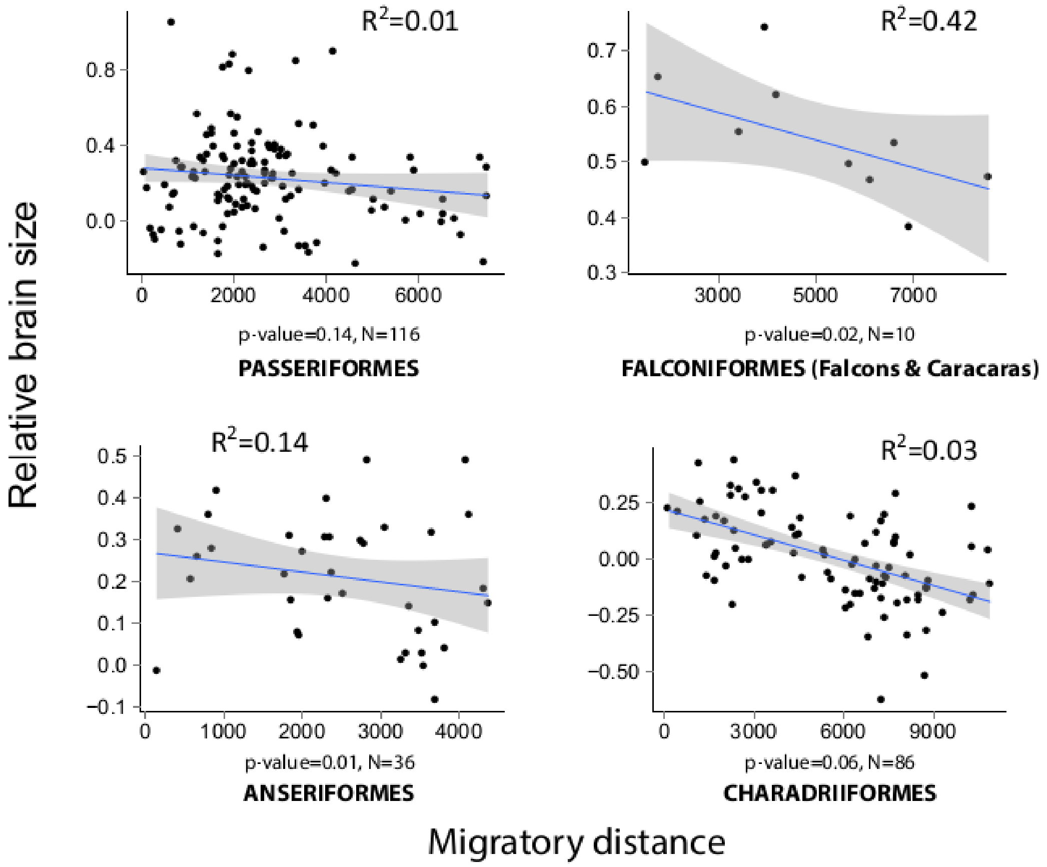

Supplementary Figure 5. Relationship between relative brain size and migratory distance within avian orders. The lines are fitted on raw data, with a SEM interval; p-values and R^2 are derived from PGLS models. Only orders with at least 10 species are presented.

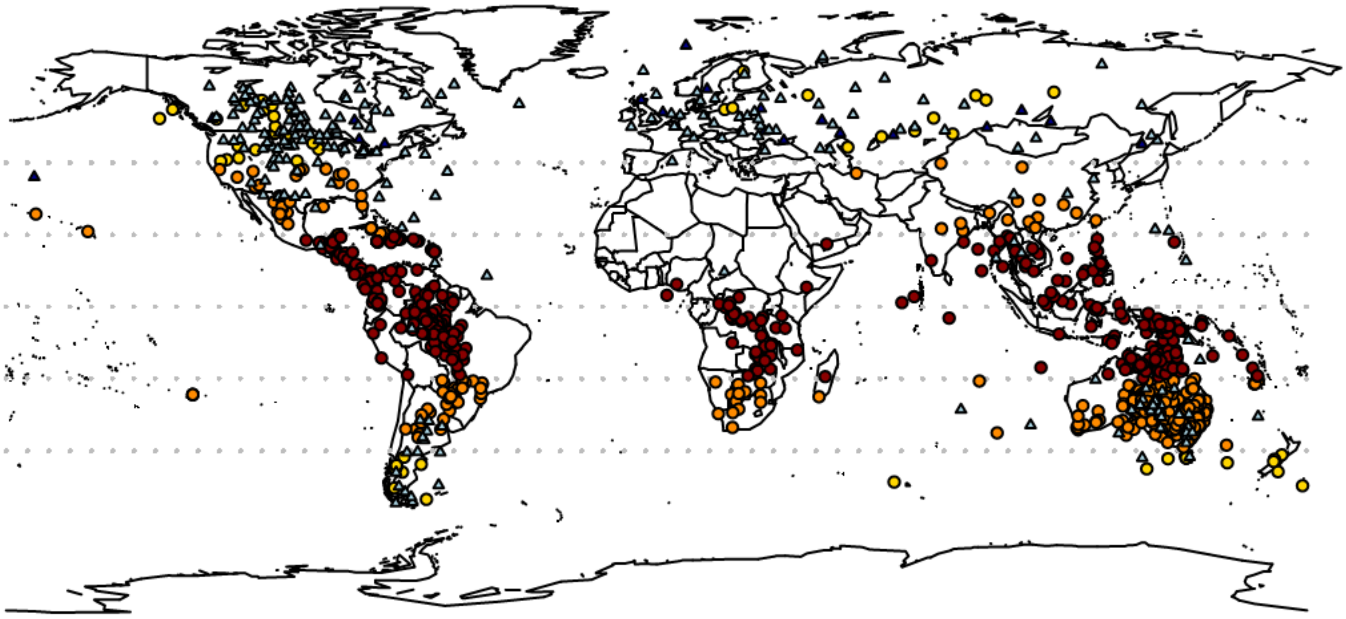

Supplementary Figure 6. Distribution of the species used in the study exposed to different degrees of seasonality, divided in those residing the entire year at higher (yellow circles), medium (orange circles) and low latitudes (red circles). An alternative strategy to avoid seasonal changes is to migrate every year to avoid harsh winters, exhibiting long-distance migrations (dark-blue triangles) or short-distance travels (light-blue triangles). Each dot is plotted in the breeding centroid of the distribution area using the *worldHires* map from 'mapdata' R-package.

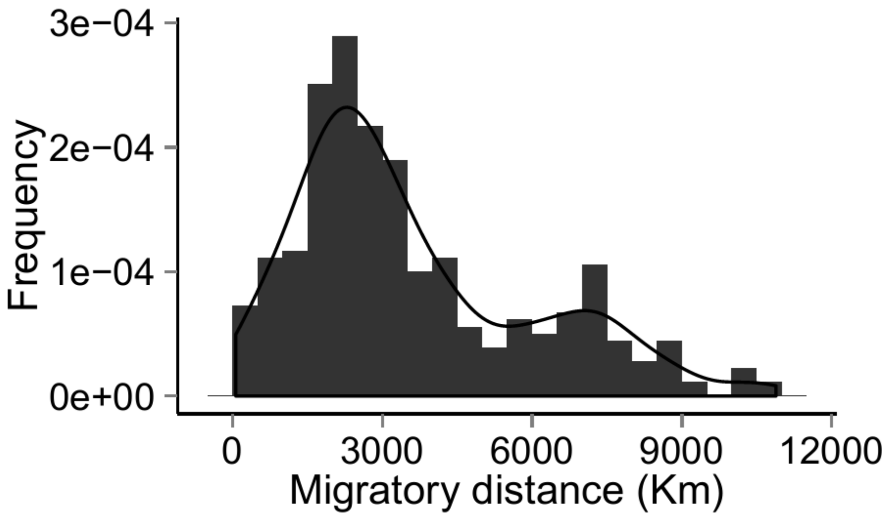

Supplementary Figure 7. Histogram of migratory distance in migratory birds (N=362 species).

a

Diet Type	PT	CH	LP	W	O
**Carrion**	16	0	5	75	4
**Fruit**	2	30.5	7	59	1.5
**Invertebrates**	17	3	5.5	70	4.5
**Nectar**	0	20.5	0	79.5	0
**Seed**	9	72	3	8	8
**Vertebrates**	16	0	5	75	4
**Plant**	4.5	34.5	3	55	3

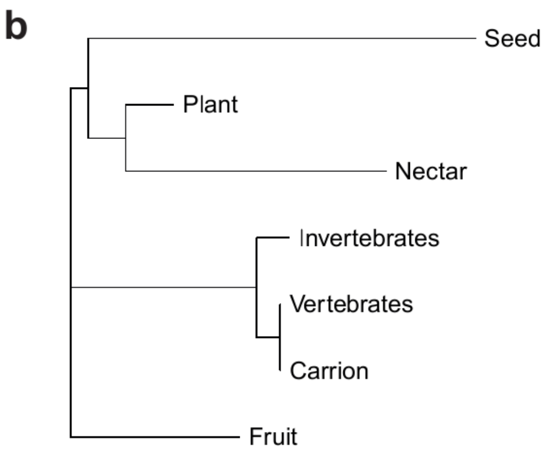

Supplementary Figure 8. Resource similarity representation used to calculate diet breadth index: (**a**) nutrient contents estimated for each food item (PT: Proteins; CH: Carbohydrates; LP: Lipids; W: Water content; O: Other), and (**b**) cluster diagram to assess similarity between diet types.

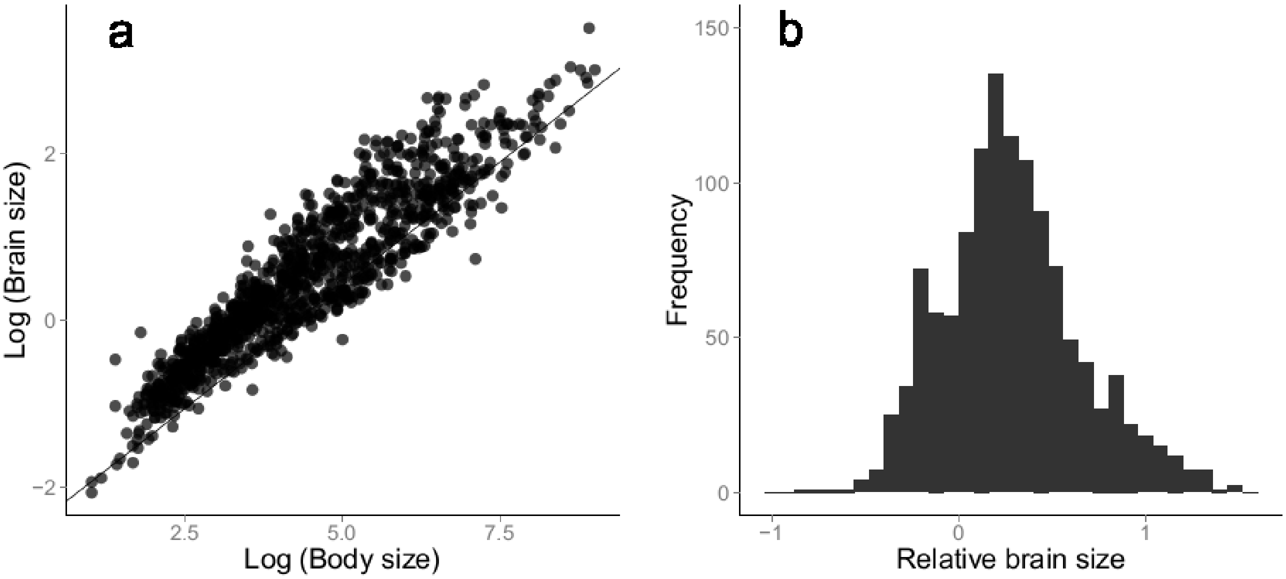

Supplementary Figure 9. Phylogenetic regression between body and brain size (**a**), used to take into account allometric effects in brain size and obtain a relative measure based on the residuals (**b**).

Supplementary Tables

Supplementary Table 1. PGLS modelling variation in brain size (log-transformed) as a function of body size and latitude (with Low latitudes taken as reference for comparisons) for resident species.

**Factor (N=855, p-value<0.001)**	**Estimate**	**SE**	**t value**	**Pr (>$\lvert t \rvert$)**	**R^2**	**λ**
Intercept	-2.62	0.17	-15.07	<0.001	0.86(0.02)	0.90
Log(body size)	0.59	0.01	73.05	<0.001		
Resident mid-latitude	0.00	0.01	0.12	0.901		
Resident high-latitude	0.06	0.03	2.44	0.015		

Supplementary Table 2. Correlation matrix for the environmental variables in resident birds (N=835). Values represent the Pearson's correlation coefficients, with the R^2 of the corresponding PGLS models with the two variables in parenthesis. The R^2 were used to calculate the variance inflator factor of the three variables (VIF=6.6).

	EVI among	EVI within
Snow	0.51 (0.25)	0.89 (0.74)
Evi within	0.53 (0.27)	-

Supplementary Table 3. PGLS modelling variation in brain size (log-transformed) as a function of body size, two environmental axis from a PPCA and including ecological categories such as fruit consumption, insect consumption and forest dwelling for resident birds.

**Factor (N=827, p-value<0.001)**	**Estimate**	**SE**	**t value**	**Pr (>$\lvert t \rvert$)**	**R^2**	**λ**
Intercept	-2.52	0.13	-19.99	<0.001	0.87(0.05)	0.91
Log(body size)	0.59	0.01	72.18	<0.001		
Environmental variation (PPC1)	0.04	0.01	5.03	<0.001		
Environmental variation (PPC2)	0.02	0.01	2.10	0.036		
Fruit consumer	0.01	0.02	0.69	0.492		
Insect consumer	0.02	0.02	0.96	0.336		
Forest dwelling	0.04	0.01	2.79	0.005		

Supplementary Table 4. PGLS modelling variation in brain size (log-transformed) as a function of body size, two environmental axes from a PPCA and diet breadth in resident birds.

**Factor (N=827, p-value<0.001)**	**Estimate**	**SE**	**t value**	**Pr (>|t|)**	R^2	**λ**
Intercept	-2.49	0.13	-19.75	<0.001	0.87(0.05)	0.92
Log(body size)	0.59	0.01	72.82	<0.001		
Environmental variation (PPC1)	0.04	0.01	5.03	<0.001		
Environmental variation (PPC2)	0.02	0.01	3.32	0.001		
Diet breadth	0.20	0.10	2.10	0.035		

Supplementary Table 5. PGLS modelling variation in brain size (log-transformed) as a function of body size, two environmental axis from a PPCA and including developmental periods (Model 1) and developmental modes (Model 2) as confound factors in resident birds.

**Factor**	**Estimate**	**SE**	**t value**	**Pr (>|t|)**	R^2	**λ**
**Model 1 (N=468, p-value<0.001)**						
Intercept	-2.75	0.23	-12.06	<0.001	0.88(0.12)	0.95
Log(body size)	0.58	0.01	42.60	<0.001		
Environmental variation (PPC1)	0.04	0.01	4.94	<0.001		
Environmental variation (PPC2)	0.04	0.01	4.11	<0.001		
Log (Incubation)	0.07	0.03	2.55	0.022		
Log (Fledging)	0.02	0.01	1.78	0.492		
**Model 2 (N=835, p-value<0.001)**						
Intercept	-2.29	0.14	-16.97	<0.001	0.87(0.07)	0.90
Log(body size)	0.59	0.01	74.01	<0.001		
Environmental variation (PPC1)	0.04	0.01	5.76	<0.001		
Environmental variation (PPC2)	0.02	0.01	3.19	0.001		
Dev.mode (Precocial)	-0.37	0.11	-3.45	0.001		
Dev.mode (Semialtricial)	0.23	0.09	2.03	0.008		
Dev.mode (Semiprecocial)	-0.15	0.12	-1.88	0.206		

Supplementary Table 6. PGLS modelling variation in brain size (log-transformed) as a function of body size, two environmental axis from a PPCA and including the social mating system (Model 1), degree of colonial breeding (Model 2) and social foraging (Model 3) as confound factors in resident birds. Monogamy is taken as the reference level in social mating system and solitary is taken as reference level in the case of colonial breeding and social foraging.

**Factor**	**Estimate**	**SE**	**t value**	**Pr (>|t|)**	**R^2**	**λ**
**Model 1 (p-value<0.001, N=590)**						
Intercept	-2.25	0.15	-14.96	<0.001	0.87(0.03)	0.91
Log(body size)	0.59	0.01	60.75	<0.001		
Environmental variation (PPC1)	0.04	0.01	4.62	<0.001		
Environmental variation (PPC2)	0.03	0.01	3.16	0.002		
Mating system (Facultative)	0.03	0.06	0.48	0.626		
Mating system (Polygamous)	-0.04	0.04	-1.17	0.242		
**Model 2 (N=443, p-value<0.001)**						
Intercept	-2.29	0.16	-14.50	<0.001	0.87(0.02)	0.97
Log(body size)	0.60	0.01	54.46	<0.001		
Environmental variation (PPC1)	0.03	0.01	3.61	<0.001		
Environmental variation (PPC2)	0.02	0.01	2.31	0.002		
Colonial Breeding (Colonial)	0.00	0.04	0.04	0.969		
Colonial Breeding (Semi-colonial)	0.00	0.03	0.12	0.904		
**Model 3 (N=302, p-value<0.001)**						
Intercept	-2.36	0.18	-13.00	<0.001	0.89(0.04)	0.95
Log(body size)	0.59	0.01	45.18	<0.001		
Environmental variation (PPC1)	0.05	0.01	4.18	0.002		
Environmental variation (PPC2)	0.04	0.01	3.12	0.001		
Social Foraging (Pairs)	0.04	0.04	0.88	0.378		
Social Foraging (Bonded Groups)	0.01	0.04	0.35	0.730		
Social Foraging (Aggregations)	0.01	0.04	0.19	0.846		

Supplementary Table 7. PGLS modelling variation in brain size (log-transformed) as a function of body size, two environmental axis from a PPCA and including ecological categories, diet breadth, developmental periods, mating system, degree of colonial breeding and social foraging as confound factors in resident birds. Monogamy is taken as the reference level in social mating system and solitary is taken as reference level in the case of colonial breeding and social foraging.

**Factor (N=242, p-value <0.001)**	**Estimate**	**SE**	**t value**	**Pr (>|t|)**	$\mathbf{R^2}$	$\boldsymbol{\lambda}$
Intercept	-2.90	0.32	-9.14	<0.001	0.87(0.43)	1.0
Log(body size)	0.58	0.02	33.01	<0.001		
Environmental variation (PPC1)	0.03	0.01	2.39	0.018		
Environmental variation (PPC2)	0.05	0.02	3.31	0.001		
Fruit consumer	-0.05	0.03	-1.69	0.092		
Insect consumer	-0.02	0.03	-0.61	0.541		
Forest dwelling	0.03	0.03	0.95	0.342		
Diet breadth	0.48	0.16	3.31	0.003		
Log (Incubation)	0.23	0.09	2.47	0.014		
Log (Fledging)	-0.01	0.01	-1.81	0.072		
Mating system (Facultative)	0.00	0.07	0.00	0.998		
Mating system (Polygamous)	0.02	0.05	0.36	0.718		
Colonial Breeding (Semi-colonial)	0.07	0.02	3.05	0.003		
Colonial Breeding (Colonial)	0.07	0.07	1.06	0.291		
Social Foraging (Pair)	0.04	0.05	0.88	0.378		
Social Foraging (Bonded group)	0.05	0.04	1.26	0.211		
Social Foraging (Aggregation)	0.03	0.04	0.84	0.406		

Supplementary Table 8. Model selection table including the beta estimate for each predictor included in the best models (increase in AICc<2) of Log(Brain size) as a response of body size, two environmental axis from a PPCA and all confound factors (N=242) with the importance in terms of AICc weight of each factor below. social mating system, colonial breeding and social foraging do not appear in the table because they do not enter in any of the best models.

Intercept	Body size	Env. var. (PPC1)	Env. var. (PPC2)	Diet Breadth	Incubation	Fledging	Forest Dweller	Fruit Consumer	Insect Consumer	df	AICc	delta	weight
-2.744	0.594	0.009	0.021	0.263	0.1508	-	-	-	-	6	-97.8	0.00	0.105
-2.703	0.597	0.008	0.021	-	0.1377	-	-	-	-	5	-97.8	0.00	0.105
-2.345	0.611	0.009	0.022	-	-	-	-	-	-	4	-97.5	0.27	0.092
-2.376	0.613	0.007	0.019	-	-	-	0.040	-	-	5	-97.5	0.32	0.09
-2.690	0.601	0.007	0.018	-	0.1226	-	0.035	-	-	6	-97.2	0.58	0.079
-2.352	0.609	0.009	0.022	0.234	-	-	-	-	-	5	-97.1	0.72	0.073
-2.729	0.598	0.007	0.019	0.254	0.1356	-	0.033	-	-	7	-97.1	0.73	0.073
-2.381	0.612	0.007	0.019	0.227	-	-	0.039	-	-	6	-96.9	0.86	0.068
-2.787	0.595	0.008	0.020	0.272	0.1685	-0.009	-	-	-	7	-96.6	1.22	0.057
-2.739	0.598	0.008	0.020	-	0.1524	-0.009	-	-	-	6	-96.4	1.36	0.053
-2.728	0.602	0.007	0.017	-	0.1379	-0.010	0.037	-	-	7	-96.0	1.78	0.043
-2.775	0.599	0.007	0.018	0.263	0.1538	-0.010	0.035	-	-	8	-96.0	1.79	0.043
-2.375	0.616	0.007	0.018	-	-	-0.007	0.042	-	-	6	-95.9	1.93	0.040
-2.715	0.598	0.009	0.021	-	0.1377	-	-	-	0.011	6	-95.8	1.98	0.039
-2.741	0.594	0.009	0.021	0.291	0.1492	-	-	-0.012	-	7	-95.8	1.99	0.039

Supplementary Table 9. PGLS modelling the EVI amplitude (difference between summer and winter in the breeding regions) as a function of latitude and migratory distance categories (with low latitudes residents as reference level to compare them with residents from other regions and with migratory birds moving short and long distances).

**Factor (N=1159)**	**Estimate**	**SE**	**t value**	**Pr (>|t|)**	**R^2**	**λ**
Intercept	-0.03	0.02	-1.72	0.085	0.25	0.35
Resident mid-latitude	0.01	<0.01	1.61	0.107		
Resident high-latitude	-0.07	0.01	-7.65	<0.001		
Migrant short-distance	-0.08	0.01	-15.93	<0.001		
Migrant long-distance	-0.08	0.01	-9.82	<0.001		

Supplementary Table 10. PGLS modelling variation in brain size (log-transformed) as a function of body size and coefficient of variation of EVI along the year (Model 1) or body size and coefficient of variation of EVI between years (Model 2) in migratory birds.

**Factor**	**Estimate**	**SE**	**t value**	**Pr (>|t|)**	**R^2**	**λ**
**Model 1 (N=317, p-value<0.001)**						
Intercept	-2.39	0.12	-20.47	<0.001	0.90(0.00)	0.88
Log(body size)	0.61	0.01	52.85	<0.001		
CV (EVI along the year)	-0.05	0.20	-0.24	0.809		
**Model (N=317, p-value<0.001)**						
Intercept	-2.38	0.12	-19.32	<0.001	0.90(0.00)	0.89
Log(body size)	0.61	0.01	52.64	<0.001		
CV (EVI among years)	-1.46	3.34	-0.43	0.666		

Supplementary Table 11. PGLS modelling variation in brain size (log-transformed) as a function of body size, latitude and migratory distance categories (with middle latitudes residents as reference level to compare them with residents from other regions and with migratory birds moving short and long distances).

**Factor (N=1217, p-value<0.001)**	**Estimate**	**SE**	**t value**	**Pr (>|t|)**	**R^2**	**λ**
Intercept	-2.48	0.12	-20.66	<0.001	0.86(0.02)	0.90
Log(body size)	0.59	0.01	86.38	<0.001		
Resident low-latitude	0.01	0.01	2.30	0.527		
Resident high-latitude	0.06	0.02	2.51	0.022		
Migrant short-distance	-0.03	0.01	-1.81	0.023		
Migrant long-distance	-0.06	0.02	-2.29	0.020		

Supplementary Table 12. PGLS modelling variation in brain size (log-transformed) as a function of body size, migratory distance and including developmental periods (Model 1) and developmental modes (Model 2) as confound factors in migratory birds.

**Factor**	**Estimate**	**SE**	**t value**	**Pr (>|t|)**	**R^2**	**λ**
**Model 1 (N=314, p-value<0.001)**						
Intercept	-2.32	0.12	-18.79	<0.001	0.90(0.13)	0.90
Log(body size)	0.59	0.01	42.93	<0.001		
Log(Migratory distance)	-0.03	0.01	-2.55	0.011		
Log (Incubation)	0.19	0.08	2.41	0.017		
Log (Fledging)	0.02	0.01	1.78	0.085		
**Model 2 (N=362, p-value<0.001)**						
Intercept	-2.27	0.12	-19.91	<0.001	0.91(0.08)	0.85
Log(body size)	0.61	0.01	56.64	<0.001		
Log(Migratory distance)	-0.03	0.01	-2.68	0.008		
Dev.mode (Precocial)	-0.32	0.09	-3.49	0.001		
Dev.mode (Semialtricial)	0.18	0.09	2.03	0.043		
Dev.mode (Semiprecocial)	-0.20	0.11	-1.88	0.061		

Supplementary Table 13. PGLS modelling variation in brain size (log-transformed) as a function of body size, migratory distance and including the social mating system (Model 1), degree of colonial breeding (Model 2) and social foraging (Model 3) as confound factors in migratory birds. Monogamy is taken as the reference level in social mating system and solitary is taken as reference level in the case of colonial breeding and social foraging.

Factor	Estimate	SE	t value	Pr (>|t|)	R^2	λ
**Model 1 (N=311, p-value<0.001)**						
Intercept	-2.13	0.16	-12.96	<0.001	0.90(0.02)	0.89
Log(body size)	0.61	0.01	50.38	<0.001		
Log(Migratory distance)	-0.03	0.01	-2.28	0.023		
Mating system (Facultative)	0.02	0.03	0.74	0.460		
Mating system (Polygamous)	-0.01	0.04	-0.13	0.897		
**Model 2 (N=296, p-value<0.001)**						
Intercept	-2.08	0.17	-12.14	<0.001	0.90(0.03)	0.89
Log(body size)	0.61	0.01	50.59	<0.001		
Log(Migratory distance)	-0.04	0.01	-2.55	0.001		
Colonial Breeding (Colonial)	-0.02	0.03	-0.50	0.618		
Colonial Breeding (Semi-colonial)	-0.04	0.02	-1.85	0.065		
**Model 3 (N=228, p-value<0.001)**						
Intercept	-2.24	0.19	-11.82	<0.001	0.91(0.01)	0.90
Log(body size)	0.60	0.01	45.76	<0.001		
Log(Migratory distance)	-0.02	0.02	-0.94	0.344		
Social Foraging (Pairs)	0.04	0.06	0.69	0.490		
Social Foraging (Bonded Groups)	-0.01	0.05	-0.17	0.862		
Social Foraging (Aggregations)	-0.03	0.03	-1.17	0.245		

Supplementary Table 14. Support for alternative models of brain size evolution (i.e. two Brownian-motion models and five Orstein-Uhlebeck models) using the mean absolute value of Akaike information criteria (AICc) and the mean Bayesian Information Criterion (BIC) to identify the best model. Models are based on 100 randomly selected phylogenies from the stochastic character mapping of different selective regimes.

	**BM1**	**BMS**	**OU1**	**OUM**	**OUMV**
**AICc**	9.12	-161.26	-160.52	-166.87	**-247.41**
**BIC**	19.31	-130.71	-145.23	-131.24	**-191.50**

Supplementary Table 15. Mean estimates and confidence intervals (2.5 and 97.5%) for the parameters (optimum, strength of selection and variation around the optimum) of the OUMV model for different selective regimes of environmental variability integrated over 100 character stochastic map trees. In the last two columns, we report in how many of the 100 trees residents from higher latitudes have higher values than other residents and long distance migrants have lower values than short distance migrants.

OUMV Model (N=100)	Res High Latitudes	Res Medium Latitudes	Res Low Latitudes	Short-distance migrant	Long-distance migrants	Models with Resident High lat. > Other residents	Models with Long-dist. migrant < Short-dist. migrant
**Brain optima (θ)**	0.68	0.19	0.19	0.06	-0.07	100 %	96 %
	(0.43/0.99)	(0.12/0.30)	(0.09/0.33)	(-0.09/0.26)	(-0.21/0.06)		
**Variation around optima (σ^2)**	0.07	0.05	0.04	0.03	0.02	76 %	1 %
	(0.00/1.35)	(0.01/0.84)	(0.01/0.83)	(0.00/0.32)	(0.02/0.47)		

Supplementary Table 16. Mean estimates and confidence intervals (2.5 and 97.5%) for the parameters (optimum, strength of selection and variation around the optimum) of the OUMVA model for different selective regimes of environmental variability integrated over 100 character stochastic map trees. In the last two columns, we report in how many of the 100 trees residents from higher latitudes have higher values than other residents and long distance migrants have lower values than short distance migrants.

**OUMVA Model (N=100)**	**Res High Latitudes**	**Res Medium Latitudes**	**Res Low Latitudes**	**Short-distance migrant**	**Long-distance migrants**	**Models with Resident High lat. > Other residents**	**Models with Long-dist. migrant < Short-dist. migrant**
**Brain optima (θ)**	0.42<br>(0.00/0.75)	0.10<br>(-0.36/0.29)	0.19<br>(-0.32/0.28)	0.05<br>(-0.31/0.20)	0.00<br>(-0.12/0.14)	81 %	70 %
**Strength of selection (α)**	0.03<br>(0.00/2.51)	0.01<br>(0.00/2.81)	0.02<br>(0.01/3.19)	0.02<br>(0.00/2.89)	0.02<br>(0.00/1.95)	79 %	33 %
**Variation around optima (σ^2)**	<0.01<br>(0.00/1.21)	<0.01<br>(0.00/0.98)	<0.01<br>(0.00/0.76)	<0.01<br>(0.00/0.49)	<0.01<br>(0.00/0.45)	17 %	30 %

Supplementary Methods

Geographical range and migratory behaviour. For each species, we extracted the information of the geographical range from BirdLife International Maps[1]. In our analysis, only distribution areas (e.g. polygons) coded as extant or probably extant were included. Non-native distribution areas (i.e. places where species have been introduced species) were excluded. Based on geographic information, we distinguished migratory from resident species on the basis of the existence of distinct breeding and non-breeding regions [2]. Therefore we use migratory category in a broader sense, partial migrants being included in this category as well[3]. However, we further refined it by estimating migratory distance, measured as the distance on the Earth surface from the breeding centroid to the non-breeding centroid. This two points (P1 and P2) on the earth surface are determined by a latitude and longitude in radiant measures: P1(*Lat1*,*Lon1*), P2(*Lat2*,*Lon2*). In Cartesian coordinates we have P1(*x1*,*y1*,*z1*) and P2(*x2*,*y2*,*z2*), where x, y and z are determined by the spherical coordinates (where R is the earth radius; R=6378 Km). Accordingly, $x = R * cos(Lat) * cos(Lon)$; $y = R * cos(Lat) * sin(Lon)$; $z = R * sin(Lat)$ and we can calculate then the Euclidean distance d between P1 and P2 by the three-dimensional Pythagorean theorem: $d = \sqrt{(x1 - x2)^2 + (y1 - y2)^2 + (z1 - z2)^2}$. This Euclidean distance was used as a proxy of the migratory distance travelled in kilometres. For partial migrants, if both breeding and wintering areas were available, the distance was calculated between these two areas. If not, the distance was calculated as the distance between resident centroid and non-breeding centroid or resident centroid and breeding centroid. We then plotted the migratory distance frequencies and we identified two clearly defined groups: birds migrating less than 5000 km and birds migrating more than 5000 km (Supplementary Fig. 7). This threshold was used to classify short and long-distance migrants. Finally, we also divided resident species into low latitudes (between 0º and 20º of latitude centroid of breeding regions), medium latitudes (between 20º and 40º) and high latitudes (above 40º of latitude). Therefore, we ended up with five categories (short-distance migrants, long-distance migrants, high latitude residents, medium latitude residents and low latitude residents) representing different selective regimes for

environmental variation (either characterized by degree of seasonality due to latitude differences or by mobility among regions). We then classified each of the 1,217 species for which we had information for brain size in one of these categories (See Supplementary Fig. 6, drawn using 'maps'[4] and 'mapdata'[5] R-packages.). Altitudinal movements and nomadic movements were not considered, so species following these movement patterns were pooled together within the category of resident species. Finally, birds that spent an important part of their life in open sea (e.g. pelagic birds) were neither considered because their migratory routes are largely unknown and the seasonality in their resources cannot be estimated using EVI information, as is the case for land species.

Environmental data. To characterise environmental variability, we used data from MODIS sensor, which was processed to generate the enhanced vegetation index (EVI)[6]. EVI is a measure derived from the normalized difference vegetation index (NDVI). Both indices use chlorophyll radiation to estimate active leave density, which is a good proxy of primary production[7]. However, EVI has improved sensitivity in high biomass regions and improved vegetation monitoring through a decoupling of the canopy background signal and a reduction in atmosphere influences. EVI index is therefore a good proxy for primary productivity over time[8,9]. We used EVI time series from the available years (2000 to 2014) at 16 days of temporal resolution and 0.05º of spatial resolution[6]. We estimated EVI of each breeding and non-breeding area using ‘sp’[10,11], ‘raster’[12] and ‘rgdal’[13], ‘rgeos’[14] R-packages[15]. This was done by intersecting the raster of environmental data with the polygons of the corresponding distribution areas of species in each period of the year. Using the 16 day product, we calculated inter-year Mean and SD for each of the 23 Julian days provided for the product along the 15 year of the temporal series. With this data, we obtained EVI annual mean, the EVI mean for breeding and non-breeding periods and the coefficient of variation (CV) of EVI among years and within years. For CV of EVI among years, we first calculated the CV for each day of the year and then computed the mean of the values. For the CV of EVI within the year, we used the mean values of EVI for each day of the year to calculate the CV. For migratory birds, we only used information from the breeding areas in the breeding season and the wintering areas in the non-breeding season, thus avoiding the period for which

migratory birds are moving. For the northern hemisphere, December-February is considered the non-breeding season and May-July is the breeding season. In the southern hemisphere we considered December-February (Julian days 337, 353, 1, 17, 33 & 49) to be the breeding period and May-July is the non-breeding season (Julian days 129, 145, 161, 177, 193 & 209). To measure the variation we used the coefficient of variation (CV hereafter) instead of the standard deviation (SD) to avoid the higher influence of the mean on the SD. When calculating the CV, we added to all values of the mean EVI the minimum value plus 1 to make sure the minimum value of the data did not included 0 or any negative value. For resident species, it was also possible to quantify the average weeks of snow cover per year over their distribution areas. For this purpose, we used MODIS snow cover series [16] from 2000 to 2014 at 1 week of temporal resolution and 0.05º of spatial resolution.

Diet breadth and other ecological factors. Birds could be affected differently by the seasonality of the environment depending on a number of factors, and these were taken into account in the analyses. First, we estimated diet breadth of each species. Each species was scored along a scale for every one of the seven different food categories: vertebrates, carrions, plants, fruits, seeds, pollen/nectar and invertebrates**.** The scale considered four possibilities: almost exclusively consumed (1), frequently consumed (0.5), rarely consumed (0.1) and not consumed (0). Categories described as 'chiefly', 'primarily', 'exclusively' or 'mainly' consumed, were given a score of 1; categories described as 'usual' or included in food items' enumerations (e.g. 'variety of foods including [enumeration of foods]', 'diet includes [enumeration of foods]') were given a score of 0.5; and categories reported as 'occasionally', 'opportunistically' or 'rarely' consumed were given a score of 0.1. We considered information at the species level, and therefore gave to each food category the maximum value reported in any of the populations of the species (e.g. if one population only eats fruits but another population of the same species eats fruits and insects, "fruits" was given a score of 1 and insect a score of 0.5). Note however that for the great majority of species, details on diet composition at the population level were not available. We then estimated diet breadth using Rao's quadratic entropy as implemented in the r-package 'indicspecies'[17,18]. This

approach estimates diet breadth as the number of categories consumed taking into account that different food items have different degrees of similarity among each other. The approach thus uses the frequency of use of each food category as well as a distance matrix of similarity between categories. Two species including two food categories in their diet might have different diet breadths, depending on how similar these categories are. For example, according to our index of diet breadth, a species that consumes two distant food categories (e.g. fruit and vertebrates) is considered as having a broader diet than another that consumes two more closely related categories (e.g. grass and seeds). The distance matrix used was calculated as a Euclidean distance (Supplementary Fig. 8) using published information on water and nutrient content (percentage of sugars, lipids, protein) of each food category: Fruits [19,20]; Plants [21,22]; Seeds [23–25]; Nectar/Pollen [26,27]; Vertebrates [28–30]; Invertebrates [30–34]. In addition to this continuous index of diet breadth, we included in our dataset two categorical factors describing the consumption of two highly seasonal resources: fruits and insects[35]. We classified each bird species as a fruit consumer (1 = consumer / 0 = not a consumer) or insect consumer (1/0). We consider a species a fruit or insect consumer if that species frequently or only eats the specific food source (1 and 0.5 scores in the diet data detailed before), but not if they consume it occasionally (0.1 score). Third, we took into account whether birds were forest dwellers or not (1/0) because this habitat might buffer seasonal changes [36]. A species was classified as a forest dweller if it regularly uses forest habitats for breeding. We considered as forest any landscape covered with trees or woody vegetation above three meters. All the information on diet and habitat uses was obtained from the Handbook of Birds of the World Alive[37].

Developmental factors. Length of the incubation and fledging periods were obtained as the number of days between laying to hatching and from hatching to fledging, respectively, and was obtained from various published sources [37–51]. Developmental mode was obtained from Iwaniuk's database[52] considering four categories (precocial, semiprecocial, altricial and semialtricial). Superprecocial species were considered precocial to reduce the number of categories. For a few species for which information

was available, we used the family value as at this taxonomic level, we found no discrepancies among species in developmental mode.

Social factors. Social mating system was obtained from published sources [37,53–60], categorizing species as monogamous or polygamous. We considered a species as socially monogamous if it raises offspring with a single mate, even if there are some levels of extra-pair paternity. Other forms of mating systems (e.g., polygyny, polyandry), were therefore considered polygamous. A third category – facultative mating system – was subsequently added for species in which both monogamy and polygamy are reported (e.g. the Boreal Owl (*Aegolius funereus*) is monogamous but there are some cases of polygyny and polyandry reported that coincide with peak periods of voles' abundance). We then defined the Colonial breeding as the degree of nest aggregation during the breeding season: we considered as colonial a species that breeds in a site with multiple nests of the same species close to each other, with no territorial defense; solitary breeders consisted in species that breed within a territory that defend from competing conspesifics. A third category, semi-colonial species, included species that breed in loose colonies (sparse aggregations of nests) or may shift between colonial and territorial breeding (e.g. the White-faced Heron *(Egretta novaehollandiae)* normally breeds solitary but sometimes breeds in colonies). All information for colonial breeding was obtained from the Handbook of the Birds of the World Alive[37]. Social foraging was defined by the degree of gregarious foraging behaviour outside of the breeding season. Solitary species were defined as those where birds forage alone. Pairs were defined as those where two reproductively mature adults continue to forage together in the non-breeding period, sometimes with other family parties. Bonded foraging groups were defined by small (<30) and stable group membership, where individuals always forage with the group. Aggregations were defined as species foraging in large numbers (>30) or by cases of highly variable social foraging, where both solitary and group foraging occur. Data for social foraging was obtained from Shultz & Dunbar[61] and completed with information from the Handbook of the Birds of the World Alive[37].

Supplementary References

1. BirdLife International & NatureServe. *Bird species distribution maps of the world*. (Available: http://www.birdlife.org/datazone/info/spcdownload, 2012).
2. Newton, I. *The migration ecology of birds*. (Academic Press, 2010).
3. Somveille, M., Manica, A., Butchart, S. H. M. & Rodrigues, A. S. L. Mapping Global Diversity Patterns for Migratory Birds. *PLoS One* **8,** e70907 (2013).
4. Original S code by Richard A. Becker and Allan R. Wilks. R version by Ray Brownrigg. Enhancements by Thomas P Minka and Alex Deckmyn. maps: Draw Geographical Maps. R package (version 3.1.1). (2016).
5. Original S code by Richard A. Becker and Allan R. Wilks. R version by Ray Brownrigg. mapdata: Extra Map Databases. R package (version 2.2-6). (2016).
6. Land Processes Distributed Active Archive Center (LP DAAC). MODIS 13C1 Vegetation Indices. Version 5. (2001). doi:http://dx.doi.org/10.5067/MODIS/MOD13C1.005
7. Phillips, L. B., Hansen, A. J. & Flather, C. H. Evaluating the species energy relationship with the newest measures of ecosystem energy: NDVI versus MODIS primary production (DOI:10.1016/j.rse.2008.04.012). *Remote Sens. Environ.* **112,** 4381–4392 (2008).
8. Fernández-Martínez, M., Garbulsky, M., Peñuelas, J., Peguero, G. & Espelta, J. M. Temporal trends in the enhanced vegetation index and spring weather predict seed production in Mediterranean oaks. *Plant Ecol.* **216,** 1061–1072 (2015).
9. Boyd, D. S., Almond, S., Dash, J., Curran, P. J. & Hill, R. A. Phenology of vegetation in Southern England from Envisat MERIS terrestrial chlorophyll index (MTCI) data. *Int. J. Remote Sens.* **32,** 8421–8447 (2011).
10. Roger S. Bivand, Edzer Pebesma, V. G.-R. *Applied spatial data analysis with R*. (Springer, 2013).
11. Pebesma, E. J. & Bivand, R. S. Classes and methods for spatial data in R. R News 5 (2). *R news* (2005).
12. Hijmans, R., Etten, J. van & Mattiuzzi, M. R Package ‘raster’. (2015).
13. Bivand, R., Keitt, T. & Rowlingson, B. rgdal: Bindings for the Geospatial Data Abstraction Library. (2016).
14. Bivand, R. & Rundel, C. rgeos: Interface to Geometry Engine - Open Source (GEOS). (2016).
15. R, C. T. R: A language and environment for statistical computing. *R Foundation for Statistical Computing* (2013). doi:ISBN 3-900051-07-0
16. Hall, D. K., Riggs, G. A. & Salomonson, V. V. MODIS/Terra Snow Cover 8-Day L3 Global 0.05deg CMG V005 [February 2000 to February 2014].

17. Cáceres, M. De, Sol, D., Lapiedra, O. & Legendre, P. A framework for estimating niche metrics using the resemblance between qualitative resources. *Oikos* 1341–1350 (2011). doi:10.1111/J.1600-0706.2011.19679.x

18. Caceres, M. De & Legendre, P. Associations between species and groups of sites: indices and statistical inference. *Ecology* (2009).

19. Foster, M. S. Ecological and Nutritional Effects of Food Scarcity on a Tropical Frugivorous Bird and its Fruit Source. *Ecol. Soc. Am.* **58,** 73–85 (1977).

20. Herrera, C. M. Vertebrate-dispersed plants of the Iberian Peninsula: a study of fruit characteristics. *Ecol. Monogr.* 305–331 (1987).

21. McLandress, M. R. & Raveling, D. G. Changes in diet and body composition of Canada geese before spring migration. *Auk* 65–79 (1981).

22. Treichler, R., Stow, R. W. & Nelson, A. L. Nutrient content of some winter foods of ruffed grouse. *J. Wildl. Manage.* **10,** 12–17 (1946).

23. Kelrick, M. I., MacMahon, J. A., Parmenter, R. R. & Sisson, D. V. Native seed preferences of shrub-steppe rodents, birds and ants: the relationships of seed attributes and seed use. *Oecologia* **68,** 327–337 (1986).

24. Kerley, G. I. H. & Erasmus, T. What do mice select for in seeds? *Oecologia* **86,** 261–267 (1991).

25. Christian, D. P. & Lederle, P. E. Seed properties and water balance in desert granivores. *Southwest. Nat.* 181–188 (1984).

26. Baker, H. G. Sugar concentrations in nectars from hummingbird flowers. *Biotropica* 37–41 (1975).

27. PYKE, G. H. The foraging behaviour of Australian honeyeaters: a review and some comparisons with hummingbirds. *Aust. J. Ecol.* **5,** 343–369 (1980).

28. Dierenfeld, E. S., Alcorn, H. L., Jacobsen, K. L. & others. *Nutrient composition of whole vertebrate prey (excluding fish) fed in zoos*. (US Department of Agriculture, Agricultural Research Service, National Agricultural Library, Animal Welfare Information Center, 2002).

29. Özogul, F., Yesim, Ö., Olgunoglu, A. I. & Boga, E. K. Comparison of fatty acid, mineral and proximate composition of body and legs of edible frog (Rana esculenta). *Int. J. Food Sci. Nutr.* **59,** 558–565 (2008).

30. Bernard, J. B. & Allen, M. A. Feeding captive piscivorous animals: nutritional aspects of fish as food. *Nutr. Advis. Gr. Handbook. Fact Sheet* **5,** (1997).

31. Oonincx, D. & Dierenfeld, E. S. An investigation into the chemical composition of alternative invertebrate prey. *Zoo Biol.* **31,** 40–54 (2012).

32. Fagbuaro, O., Oso, J. A., Edward, J. B. & Ogunleye, R. F. Nutritional status of four species of giant land snails in Nigeria. *J. Zhejiang Univ. Sci. B* **7,** 686–689 (2006).

33. Deblauwe, I. & Janssens, G. P. J. New insights in insect prey choice by chimpanzees and gorillas in southeast Cameroon: the role of nutritional value. *Am.*

J. Phys. Anthropol. **135,** 42–55 (2008).

34. Kinyuru, J. N., Kenji, G. M., Muhoho, S. N. & Ayieko, M. Nutritional potential of longhorn grasshopper (Ruspolia differens) consumed in Siaya District, Kenya. *J. Agric. Sci. Technol.* **12,** 1–24 (2010).

35. Carnicer, J., Abrams, P. a. & Jordano, P. Switching behavior, coexistence and diversification: comparing empirical community-wide evidence with theoretical predictions. *Ecol. Lett.* **11,** 802–808 (2008).

36. Chesser, R. T. & Levey, D. J. Austral migrants and the evolution of migration in new world birds: diet, habitat, and migration revisited. *Am. Nat.* **152,** 311–319 (1998).

37. Del Hoyo, J., Elliot, A. ., Sargatal, J., Christie, D. A. & de Juana, E. *Handbook of the Birds of the World Alive*. (Lynx Edicions, 2016).

38. Cassey, P. *et al.* Why are birds ' eggs colourful ? Eggshell pigments co-vary with life-history and nesting ecology among British breeding non-passerine birds. 657–672 (2012).

39. Bennett, P. M. Comparative studies of morphology, life history and ecology among birds. (University of Sussex, 1986).

40. Figuerola, J. & Green, A. J. A comparative study of egg mass and clutch size in the Anseriformes. *J. Ornithol.* **147.1,** 57–68 (2006).

41. Pienaar, J., Ilany, A., Geffen, E. & Yom-Tov, Y. Macroevolution of life-history traits in passerine birds: adaptation and phylogenetic inertia. *Ecol. Lett.* **16,** 571–6 (2013).

42. Scheuerlein, A. & Ricklefs, R. E. Prevalence of blood parasites in European passeriform birds. *Proc. Biol. Sci.* **271,** 1363–70 (2004).

43. Huang, Z. Y. X. *et al.* Species' life-history traits explain interspecific variation in reservoir competence: a possible mechanism underlying the dilution effect. *PLoS One* **8,** e54341 (2013).

44. Clark, M. E. & Martin, T. E. Modeling tradeoffs in avian life history traits and consequences for population growth. *Ecol. Modell.* **209,** 110–120 (2007).

45. Jouventin, P. & Dobson, F. S. Why breed every other year? The case of albatrosses. *Proc. Biol. Sci.* **269,** 1955–61 (2002).

46. Badyaev, A. V. & Ghalambor, C. K. Evolution of life histories along elevational gradients: trade-off between parental care and fecundity. *Ecology* **82,** 2948–2960 (2001).

47. Galván, I., Erritzøe, J., Karadaş, F. & Møller, A. P. High levels of liver antioxidants are associated with life-history strategies characteristic of slow growth and high survival rates in birds. *J. Comp. Physiol. B.* **182,** 947–59 (2012).

48. Ricklefs, R. E. Intrinsic aging-related mortality in birds. *J. Avian Biol.* **31,** 103–111 (2000).

49. Tacutu, R. *et al.* Human Ageing Genomic Resources: Integrated databases and

tools for the biology and genetics of ageing. *Nucleic Acids Res.* **41,** D1027–D1033 (2013).

50. Szekely, T., Catchpole, C. K., Devoogd, A., Marchl, Z. & Devoogd, T. J. Evolutionary changes in a song control area of the brain (HVC) are associated with evolutionary changes in song repertoire among European warblers (Sylviidae). *Proc. R. Soc. London. Ser. B Biol. Sci.* **263,** 607–610 (1996).
51. Lloyd, J. D. & Martin, T. E. Sibling competition and the evolution of prenatal development rates. *Proc. Biol. Sci.* **270,** 735–40 (2003).
52. Iwaniuk, A. N. & Nelson, J. E. Developmental differences are correlated with relative brain size in birds: a comparative analysis. *Can. J. Zool.* **81,** 1913–1928 (2003).
53. Shultz, S. & Dunbar, R. Encephalization is not a universal macroevolutionary phenomenon in mammals but is associated with sociality. *Proc. Natl. Acad. Sci. USA* (2010). doi:10.1073/pnas.1005246107/-/DCSupplemental.www.pnas.org/cgi/doi/10.1073/pnas.1005246107
54. García-Peña, G. E., Sol, D., Iwaniuk, a N. & Székely, T. Sexual selection on brain size in shorebirds (Charadriiformes). *J. Evol. Biol.* **26,** 878–88 (2013).
55. Liker, A. & Székely, T. MORTALITY COSTS OF SEXUAL SELECTION AND PARENTAL CARE IN NATURAL. *Evolution (N. Y).* **59,** 890–897 (2005).
56. Cramp, S., Simmons, K. E. L. & Perrins, C. M. *Complete birds of the Western Palearctic CD-Rom*. (Oxford University Press, 1998).
57. Rubolini, D. Protandry and sexual dimorphism in trans-Saharan migratory birds. *Behav. Ecol.* **15,** 592–601 (2004).
58. Poole, A. The Birds of North America Online. Cornell Laboratory of Ornithology, Ithaca, NY. (2005).
59. Johnsgard, P. A. *The plovers, sandpipers, and snipes of the world*. (University of Nebraska Press Lincoln, 1981).
60. Lenington, S. Female choice and polygyny in redwinged blackbirds. *Anim. Behav.* **28,** 347–361 (1980).
61. Shultz, S. & Dunbar, R. I. M. Social bonds in birds are associated with brain size and contingent on the correlated evolution of life-history and increased parental investment. *Biol. J. Linn. Soc.* **100,** 111–123 (2010).
